# Documenting the Food Insecurity Experiences and Nutritional Status of Women in India: Study Protocol

**DOI:** 10.3390/ijerph17113769

**Published:** 2020-05-26

**Authors:** Fiona H McKay, Preethi John, Alice Sims, Gaganjot Kaur, Jyotsna Kaushal

**Affiliations:** 1 School of Health and Social Development, Deakin University, Geelong, Victoria 3125, Australia; aasims@deakin.edu.au; 2 Chitkara School of Health Sciences, Chitkara University, Punjab Rajpura, Distt 140401, India; preethi@chitkara.edu.in; 3 Chitkara Business School, Chitkara University Punjab, Rajpura, Distt 140401, India; gaganjot.kaur@chitkara.edu.in; 4 Centre of Water Sciences, Chitkara University Institute of Engineering and Technology, Chitkara University, Punjab, Rajpura, Distt 140401, India; jyotsna.kaushal@chitkara.edu.in

**Keywords:** food security, India, women, rural

## Abstract

Background: Despite significant growth and change in India over the past two decades, some public health indicators have failed to keep pace. One such indicator is food insecurity. India is home to the largest number of people experiencing hunger and food insecurity. Food security is described as “a situation that exists when all people, at all times, have physical, social and economic access to sufficient, safe and nutritious food that meets their dietary needs and food preferences for an active and healthy life”. While there has been considerable research investigating the role of crop yields, policy interventions, and food production in alleviating food insecurity in India, there is insufficient research investigating the social and cultural influences of food insecurity, including the role of women. The primary aim of this research is to investigate the experience of food insecurity among women in India. The objectives of this research are (1) to determine the role of women in food production and its contribution to household food security; (2) to examine the gender roles within households and the decision-making processes that influence food security, and (3) to investigate household nutritional status and food insecurity experience. Methods: Participants will include women who live in a village in Punjab, India. Interviews with 100 households, drawn from a convenience sample will be conducted. Interviews will be conducted in Punjabi with simultaneous English translation, and will include: food related experiences, anthropometric measurements (weight, height, waist, and hip) and dietary assessment (24-h diet recall, two non-consecutive days), dwelling facilities, agriculture related information, including household agriculture activities undertaken, food security status (via the United States Department of Agriculture Household Food Security Scale Measurement), and demographic information. Discussion: This study aims to investigate a range of determinants of food insecurity among a rural population. It will allow for the identification of some of the components of household food insecurity among women in India and will go part of the way to understanding how and why India continues to experience food and nutritional insecurity despite growth and progress in a range of other indicators.

## 1. Background

Over the last two decades, India has undergone significant economic growth and change, including a substantial increase in gross domestic product and per capita income. In this time, over 60 million people have benefited from improved living conditions and have been lifted out of poverty [1]. When considering the statistics for India as a whole, there have been vast improvements in access to water and sanitation, education, health care, and workforce participation, however, some public health indicators have failed to keep pace with this change. For example, life expectancy at birth, and infant and maternal mortality rates are below the global average and the average for the South East Asian region, and over one quarter of India’s population continues to live below the poverty line, or on less than US $1.25 per day [2,3,4]. While there has been an improvement in the mortality rate for children under five years, declining to 39 per 1000 live births, a rate that matches the global rate, it is still much higher than comparable countries, including China which has an under five mortality rate of 9 per 1000. Furthermore, the level of stunting (height for age) and wasting (weight for height) continues to be very high, with 37.9% of children under five stunted in India—compared with 21.9% globally and 31.9% in the South Asian Region [4,5]. The situation for women is also slow to improve; over one third of women of reproductive age have been found to be anaemic [6], malnutrition remains high [7], and few women have control over their family or personal finances [8], all of which are contributors to food and nutritional insecurity.

The concept of food security has evolved significantly over the past few decades. The right to food can be found in Article 25 of the Universal Declaration of Human Rights (UDHR), accepted by 58 Member States of the United Nations (UN) in 1948. Article 25 proclaims the right to an adequate standard of living, including the right to food. The right to food is also included in Article 11 of the International Covenant on Economic, Social and Cultural Rights (ICESCR). While the inclusion of the right to food within these documents is important, neither, now ratified by almost all Member States of the UN, define what the right to food is, or how food security can be achieved. 

A more practical application of the right to food has been addressed through a series of World Food Summits, convened by the Food and Agriculture Organization (FAO) of the UN. In 1974, the first World Food Summit proclaimed food security as the “availability at all times of adequate world food supplies of basic foodstuffs to sustain a steady expansion of food consumption and to offset fluctuations in production and prices” [9]. Since this time, hundreds of definitions of food security have been developed [10]. The most common definition of food security describes food security as “a situation that exists when all people, at all times, have physical, social and economic access to sufficient, safe and nutritious food that meets their dietary needs and food preferences for an active and healthy life” [11]. This definition recognises the importance of the “four pillars” of food security: availability, access, utilization, and sustainability. Availability refers to the volume of food available from one’s own production, the market, or from aid, and can be impacted by a lack or problems with storage, the negative impacts of climate change, and harvest manpower. According to Barrett [12], while food availability is insufficient to ensure access to and utilization of food to achieve food security, aggregate availability is a necessary condition for food security. The second pillar, access, refers to the resources and purchasing power of a household as well as the policy environment that dictates trade, subsidies, and conflict. Access is said to reflect the demand side of food security, as well as emphasising problems relating to unexpected shocks, including natural disasters, unemployment, poor health, or price hikes [12]. Utilization, the third pillar, refers to an individual’s biological ability to use the food consumed, and considers the absence of parasites, disease, and the ability to absorb micronutrients, as well as the presence of appropriate cooking facilities and cooking skill. Finally, sustainability refers to market stability, both local and global. These pillars are hierarchical, where availability is necessary, but does not ensure access, which in turn is necessary but not sufficient to ensure utilisation, which is necessary but cannot guarantee sustainability [12,13]. 

Much of the research that has investigated food security in India has focused on the availability pillar of food security, largely through investigations of crop yields [14], policy interventions [15], and food production [16]. Research that has investigated the experience of food insecurity at the household level has mostly occurred through data collected via the National Sample Survey, consumer expenditure data, and national grain and rice production estimates [17,18]. This focus on availability, particularly the focus on purchasing power, is problematic as it emphasises the role of the distribution of government aid as a means to alleviate food insecurity, ignoring the other pillars of food insecurity. This focus on re-distribution also removes the role of any local customs, individual strategies, and social factors that contribute to food insecurity, including poverty, resulting from low and irregular incomes [19], inadequate housing and basic infrastructure [20], overcrowding and a lack of basic sanitary services resulting in the promotion of infectious diseases [21], unequal distribution of wealth and access and entitlement to land, and limited access to services [22]. This has meant that much of the formal response to chronic and acute hunger in India to date has been through activities directed at the availability pillar of food security; through food supply activities, including the implementation of the National Food Security Act of 2013 [23] and the Public Distribution System [24]. These are positive steps in responding to a problem of food shortage that has impacted many millions of people, but there is a potential that these strategies have met their capacity to make positive change; additional approaches are now required that move beyond food availability. 

There are approximately 800 million hungry people unevenly distributed across the world; two thirds live in South Asia and Africa south of the Sahara [25]. With more than 200 million food insecure people, India alone is home to the largest number of hungry people, where poor nutrition causes nearly half of all deaths in children under five [25]. India ranks 102nd of 117 countries included in the Global Hunger Index, the tool used to measure and compare hunger across developing nations [26]. While the number of hungry and malnourished in India has remained relatively stable over the past two decades [26], the emerging problem of overweight and obesity is becoming more of a concern, with recent research suggesting that there are over 135 million obese people in India [27].

The reasons for India’s chronic food insecurity, the high rates of stunting and wasting, and the emerging problems related to obesity are complex. While supply and access to food is important, undernutrition and hunger persist in India not because of problems in food production [28], but because of the interplay between the social determinants of health and inequalities in entitlement and access within Indian society [29,30]. For example, more than 30% of rural households are landless [31], relying on manual, casual labour for a large portion of their income [32], discrimination by caste, religion, and gender remain pervasive [33], and low literacy and poor formal education restrict opportunities for social mobility [34,35]. 

Women are known to disproportionately experience food insecurity. This experience of food insecurity is related to inequalities in land ownership, lingering cultural barriers to employment despite increasing education attainment [8], and the invisibility of women’s economic contribution to agriculture. While research investigating food insecurity in India is increasing, there remain gaps in our understanding of how women respond to, and cope with, food insecurity and hunger. What is also missing from current work is an investigation of the different experiences of food insecurity within households, and the role of women in protecting their families, especially elderly relatives and children, against food insecurity and hunger. 

The primary aim of this research is to investigate the experience of food insecurity among women in India. The objectives of this research are (1) to determine the role of women in food production and its contribution to household food security, (2) to examine the gender roles within households and the decision-making processes that influence food security, and (3) to investigate household nutritional status and food insecurity experience. 

## 2. Methods/Design

### 2.1. Sample and Recruitment

Participants will include women who live in the village of Thuha, located in the state of Punjab in north India. Thuha consists of approximately 2500 individuals in 500 dwellings [36]. This research will consist of interviews with 100 households, drawn from a convenience sample. Households of any size and type will be included, with the preference of interviewing women, all participants will be over 18 years of age. The preference for women is consistent with some emerging work from India that suggests that women have a large influence on the food security status of households, particularly in households where men temporarily migrate into the cities for work [37].

### 2.2. Data Collection

The village Sarpanch, the official elected head of the village, will be informed of the research and will meet with the research team before any research activity begins. With the consent of the Sarpanch, information about the study objectives and data collection requirements, including how long the survey will take, and the measurements needed, will be provided to each household. Informed verbal consent will then be obtained from the participant. Households and individuals will not be pressured to participate; all households will only be approached once for their participation. 

Data will be collected through face-to-face structured interviews either at the house of the participant or at a mutually agreed on public location. Interviews will include: food related experiences, anthropometric measurements (weight, height, waist, and hip) and dietary assessment (24-h diet recall, two non-consecutive days), dwelling facilities, agriculture related information, including household agriculture activities undertaken, food security status (via the USDA Household Food Security Scale Measurement), and demographic information. Households will be visited up to three times, and in total interviews are expected to last for approximately 30–60 min. Interviews will be audio recorded, with recordings used to supplement notes (see  Supplementary materials) taken during the interview. Interviews will be conducted in Punjabi with at least one translator fluent in English and Punjabi present to provide simultaneous translation to allow for any probing or for follow-up questions at the time of the interview. Four major domains will be explored in the interview (see Table 1).

Interviews will consist of 39 close-ended questions, and five open-ended questions. Four major domains will be explored in the interview:

1. Socioeconomic variables: this section includes close-ended questions about household type, household composition, household size, education attainment, employment, and agricultural activity.
2. Food security: a four-item version of the short-form six-item United States Department of Agriculture, Household Food Security Survey Module, developed by Blumberg and Bialostosky [38] will be used. This validated scale assesses whether participants are able to afford enough food to eat, includes questions around quantities of food eaten, the frequency of meals and food affordability. The four items that will be included in this study are:
  (a) The food that [I/We] bought just didn’t last, and [I/We] didn’t have money to get more. Was that often, sometimes, or never true for you in the last month?
  (b) [I/We] couldn’t afford to eat balanced meals. Was that often, sometimes, or never true for you in the last month?
  (c) In the last month, did you (or other adults in your household) ever cut the size of your meals or skip meals because there wasn’t enough money for food? Was that almost every week, some weeks but not every week, in only 1 or 2 weeks, or never?
  (d) In the last month, were you ever hungry but didn’t eat because you couldn’t afford enough food? (Yes, No).

Consistent with the method proposed by Agarwal and Sethi [39], questions 1 and 2 will be coded as 0 for “never true” and 1 for “sometimes true/often true”. Question 3 will be coded as 1 “almost every week, some weeks but not every week, in only 1 or 2 weeks” and 0 for “never true”. Question 4, with response options “yes” or “no” will be coded as 1 and 0, respectively. Households with zero or one affirmative response will be classified as food secure, those with two or more affirmative responses will be classified as food insecure and those with three or more affirmative responses will be classified as food insecure with hunger. 

In addition, the interview will include questions regarding how the household sources food, how often they eat, if they consider their eating patterns to be different now than they were in the past, and who makes household decisions.

3. Anthropomorphic measurements allow for easy and non-invasive determination of nutritional status. Two individual measures of women from households will be included in this study. Measurements will be taken by trained research assistants.
  (a) Body mass index (BMI), employing height and weight measures, has been found to be related to an individual’s food consumption patterns [40] and health outcomes [41,42]. Food insecurity in women often presents as high BMI [43], with some studies suggesting that the prevalence of overweight among women increases as food insecurity increases [44,45]. Participants will be weighed, without shoes, on a portable Tanita digital scale to the nearest 0.1 kg. Height will be taken with a portable Seca 213 stadiometer to the nearest 0.5 cm. All measurements will be taken twice, and the average used for calculation. BMI will be calculated as weight (kg) divided by height (m) squared (kg/m^2^). Criteria used to define overweight will be those adopted by India which considers obesity when BMI ≥ 25 kg/m^2^ [46].
  (b) Waist-to-hip ratio has been identified as a predictor of poor health outcomes, particularly cardiovascular disease [47]. Waist circumference (cm) will be taken with a Seca tape measure at the point midway between the costal margin and iliac crest in the mid-axillary line, with the subject standing and breathing normally [48]. Hip circumference (cm) will be measured at the widest point around the greater trochanter. The waist-to-hip ratio will be calculated as the waist measurement divided by the hip measurement. Given that Asian populations have been found to have an increased risk at lower waist circumference than Europeans, the lower recommended waist circumference and waist-hip ratios of 80 cm and 0.80 will be used for the women in this study [49]. Waist and hip measurements are being employed in this study as they provide more accurate information about accumulation and distribution of fat in the body than BMI alone [50].
4. The 24-h diet recall is a structured interview that will be used to capture detailed dietary information regarding all foods and beverages consumed by the respondent, including the quantities and methods of preparation within the designated 24-h period. Women are typically responsible for food preparation and best suited to provide information pertaining to the dietary diversity of their households [51]. As such, women will be the focus of the 24-h diet recall data collection. 24-h diet recall will follow the multi-pass method with reference photos, which have been shown to allow for a more comprehensive recall [52].

## 3. Data Analysis

A variety of data analysis approaches will be employed to interrogate the data collected. Basic descriptive statistics, including frequencies, percentages, means, and standard deviations will be used to investigate the quantitative data. Standard calculations of food insecurity described above will be applied to determine household food security status. The 24-h diet recall data will be entered into Foodworks 10 for nutritional analysis. Data analysis will be informed by thematic analysis following the process described by Miles and Huberman [53]. A thematic analysis, following an inductive approach, allows for the identification of themes in the data. The constant comparative method will be employed as soon as the data is collected. This technique allows for the identification of patterns and ideas in the data. The data will be read and re-read to identify codes, categories, and themes.

The study protocol has been approved by the Deakin University Research Ethics Committee (2020-007). Informed consent procedures described above allow individual participants to decline to participate. This study was funded by an Australia–India Strategic Research Fund Early- and Mid-Career Researcher 2020 Fellowship. 

## 4. Discussion

Given the severity of the food insecurity and hunger problem in India, researchers need to start to look at alternative means beyond production and distribution as solutions. To do this, measurements beyond crop production need to be considered, with investigation of household experiences of food security and hunger needed. This study will provide some insight into the role of women in rural households relating to food security and will explore some of the important cultural and gendered influences that can impact food insecurity. By including key anthropomorphic measures, the physical impacts of food insecurity among this population can be explored, while the inclusion of the 24-h diet recall will allow for an exploration of diet diversity and general nutritional intake. 

The focus of this research is to reframe current understandings of food security, with the overarching goal of contributing to knowledge that will see the reduction of the intergenerational negative health and social effects of chronic undernourishment that result from food insecurity. This will be achieved by focusing on how those in need can access a reliable, affordable, and sufficient diet, that is both nutritious and culturally appropriate. In addition, as food security is interrelated to livelihoods and entitlements [19], education [54], land [55], and engagement with the formal financial sector [56], the role of these social determinants of health will also be explored. 

## 5. Conclusions

This study will allow for the identification of some of the components of household food insecurity among women in India and will go part of the way to understanding how and why India continues to experience food and nutritional insecurity despite growth and progress in a range of other indicators.

## Figures and Tables

**Table 1:** Overview of four domains explored in the interviews.

Domains Explored in Interviews	Example Items to be Explored
Socioeconomic variables	Household type, composition, and size
	Education attainment
	Employment
	Agricultural activity
Food security	Not enough money for food
	Could not afford to eat balanced meals
	Cut the size of meals
	Experience hunger
	Meals per day
	Food storage
	Food costs
Anthropomorphic measurements	Height (cm)
	Weight (kg)
	Waist circumference (cm)
	Hip circumference (cm)
24-h recall	All foods consumed in the past 24-h period

## Supplementary Materials

The following are available online at https://www.mdpi.com/1660-4601/17/11/3769/s1, The survey used in this study will be made available as a supplementary file.

## Declarations

Ethics approval and consent to participate in this project was obtained from the Deakin University Human Research Ethics Committee (ref: 2020-007). All participants will be provided with a statement to describe the purpose of the study and will provide verbal consent.

## Abbreviations

BMI	Body Mass Index
FAO	Food and Agriculture Organization
ICESCR	International Covenant on Economic, Social and Cultural Rights
UDHR	Universal Declaration of Human Rights
UN	United Nations
