# Supplementary material for: Documenting the Food Insecurity Experiences and Nutritional Status of Women in India: Study Protocol
Source: Int J Environ Res Public Health. 2020 May 26;17(11):3769. doi: 10.3390/ijerph17113769 (PMC7312776; doi:10.3390/ijerph17113769)
Supplement: Supplementary file 1 [file ijerph-17-03769-s001.pdf]

Protocol

Documenting the Food Insecurity Experiences and Nutritional Status of Women in India: Study Protocol

Fiona H McKay [1,*], Preethi John [2], Alice Sims [1], Gaganjot Kaur [3] and Jyotsna Kaushal [4]

[1] School of Health and Social Development, Deakin University, Geelong, Victoria 3125, Australia; aasims@deakin.edu.au
[2] Chitkara School of Health Sciences, Chitkara University, Punjab Rajpura, Distt 140401, India; preethi@chitkara.edu.in
[3] Chitkara Business School, Chitkara University Punjab, Rajpura, Distt 140401, India; gaganjot.kaur@chitkara.edu.in
[4] Centre of Water Sciences, Chitkara University Institute of Engineering and Technology, Chitkara University, Punjab, Rajpura, Distt 140401, India; jyotsna.kaushal@chitkara.edu.in
* Correspondence: fiona.mckay@deakin.edu.au

Received: 1 May 2020; Accepted: 21 May 2020; Published: 26 May 2020

1. Phone number
2. Age of respondent
3. Age of head of household
4. Gender of head of household
5. Religion
6. Cast
7. Number of people living in house
8. Type of family
9. How many children has the respondent given birth to?
10. Number of people below 18 years
11. Number of people below 5 years
12. Approximate monthly household income
13. Education level of the Father
14. Education level of the Mother
15. Occupation of the Father
16. Occupation of the Mother
17. Own land that can be farmed? If yes, for what?
18. Own
    a. TV
    b. Radio
    c. Mobile phone

    d. Other__________

19. Transportation
    a. Car
    b. 2-wheeler
    c. Bicycle
20. Running water that can be drunk
21. Toilet
22. Electricity
23. Do you have a BPL card?
24. In the last 30 days, did you get any benefit from the PDS shop? If so, what?
25. Do you have health insurance?
26. Do you have any other insurance?
27. Do you have a bank account? If yes, does it have any deposits?
28. Does the family have any loans now?
29. Have any members of the household been to a hospital in the past 30 days? If yes, what for? Can they describe the experience?
30. Have any members of the household had diarrhea or vomiting in the past 5 days? If yes, were they treated? Are they ok now?
31. Have any members of the household been diagnosed with diabetes? If yes, are they on medications?
32. Have they ever tried to lose weight? OR to put on weight?
33. Were children in the house breastfed? If yes, for how long?
34. Does any family member consume alcohol? If yes, how much and how often?
35. Does any family member consume tobacco? If yes, what type and how many?

36. The food that we bought just didn't last, and we didn't have money to get more
    a. Was that often, sometimes, or never true for you in the last 1 month?
37. We couldn't afford to eat balanced meals.
    a. Was that often, sometimes, or never true for you in the last 12 months? Balanced meals may contain starchy food such as rice, potato, bread and wheat; and a protein-rich food such as meat/ fish/milk/curd, pulses and fruits and vegetables.

38. In the last month, did you (or other adults in your household) ever cut the size of your meals or skip meals because there wasn't enough money for food?
    a. Was that almost every month, some months but not every month, in only 1 or 2 months, never?
39. In the last month, were you ever hungry but didn't eat because you couldn't afford enough food? (Yes, No)
40. Have you heard the word “malnutrition”? If yes, what does it mean to you?

41. *Interviewer:* – If Yes or Maybe/ Not Sure, ask the meaning (please do not read from the list)
    a. Link between food and nutrition
    b. Balanced diet
    c. Adequate food
    d. Nutritious food
    e. Breastfeeding
    f. Safe water
    g. Hygiene and cleanliness
    h. Other _________
    i. Don’t know
42. Where do you get your food from?
43. How often do you need to get food?
44. How many meals per day do you eat?
45. Do you think eating patterns nowadays are healthier than those in the past?
46. How do you store your food?
47. Do you buy tinned or packaged foods?
48. Approximately how much do you spend on food each week?
49. Does your family have enough money for the food that you need?
50. Are there things that you would like to eat, but you cant afford to? If yes, what?
51. How often do you purchase food from vendors?

52. Usually, who makes the decision about:
53. Child welfare (e.g. food, clothing, health, etc.)
54. Major household purchases (e.g. TV, 2-wheeler, etc.)
